# Effect of trelagliptin on vascular endothelial functions and serum adiponectin level in patients with type 2 diabetes: a preliminary single-arm prospective pilot study

**DOI:** 10.1186/s12933-016-0468-4

**Published:** 2016-11-04

**Authors:** Satoshi Ida, Kazuya Murata, Katunori Betou, Chiaki Kobayashi, Yuki Ishihara, Kanako Imataka, Akihiro Uchida, Kou Monguchi, Ryutaro Kaneko, Ryoko Fujiwara, Hiroka Takahashi

**Affiliations:** 1 Department of Diabetes and Metabolism, Ise Red Cross Hospital, 1-471-2 Funae, 1-Chome, Ise-shi, Mie 516-8512 Japan; 2 Department of Clinical Laboratory, Ise Red Cross Hospital, 1-471-2 Funae, 1-Chome, Ise-shi, Mie 516-8512 Japan

**Keywords:** Type 2 diabetes mellitus, Dipeptidyl peptidase-4 inhibitor, Trelagliptin, Vascular endothelial functions

## Abstract

**Background:**

Trelagliptin, an oral DPP-4 inhibitor, which is administered once per week and characterized by a long half-life in blood. The effects of trelagliptin on vascular endothelial functions have not been clarified to date. The objective of the present study was to examine the effects of trelagliptin on vascular endothelial functions in patients with type 2 diabetes mellitus (DM) using flow-mediated dilatation (FMD), adiponectin, and asymmetric dimethylarginine (ADMA) as evaluation indicators.

**Methods:**

This study was a preliminary single-arm prospective pilot study. The subjects of this study were type 2 DM patients aged 20–74 years, who visited our outpatient department. The patients were treated with trelagliptin, and their FMD, adiponectin, and ADMA levels were measured at baseline and at 12 weeks after initial treatment to determine the changes during the study period.

**Results:**

A total of 27 patients, excluding three dropouts, were included in the population for analysis. Trelagliptin treatment showed no significant changes in FMD (2.42 ± 2.7% at baseline vs. 2.66 ± 3.8% post-treatment, P = 0.785) and ADMA (0.41 ± 0.0 µg/mL at baseline vs. 0.40 ± 0.0 µg/mL post-treatment, P = 0.402). Trelagliptin treatment resulted in a significant increase of serum adiponectin level (7.72 ± 6.9 µg/mL at baseline vs. 8.82 ± 8.3 µg/mL post-treatment, P < 0.002).

**Conclusions:**

In this pilot study, trelagliptin treatment showed no significant changes in FMD. On the other hand, it was believed that trelagliptin treatment may increase serum adiponectin level.

*Trial Registration* http://www.umin.ac.jp (Trial ID UMIN000018311)

## Background

The incidences of type 2 diabetes mellitus (DM) have been increasing during recent years and are associated with increasing incidences of cardiovascular diseases, which comprise a serious potential outcome [1]. Therefore, a key objective of type 2 DM management is the prevention of cardiovascular diseases. It has been suggested that the onset of cardiovascular diseases is associated with decreased arterial dilatation [2]. Flow-mediated dilatation (FMD) is extensively used to evaluate arterial dilatation [3]. Decreased arterial dilatation is triggered by a decline in nitric oxide (NO) production [4]. Some researchers have reported a decrease in NO production or its activity in DM patients [5].

In recent years, antidiabetic drugs with new mechanisms of action have become available. Of these, incretin preparations [e.g., dipeptidyl peptidase-4 (DPP-4) inhibitors and glucagon-like peptide-1 (GLP-1) analogs] improve FMD; this improvement is mediated by NO production or activation as well as effects such as the promotion of insulin secretion and inhibition of glucagon [6].

Trelagliptin is now considered as an oral DPP-4 inhibitor administered once per week and characterized by a long half-life in the blood. However, the effects of trelagliptin on vascular endothelial functions have not been clarified to date. The objective of the present study was to examine the effects of trelagliptin treatment on vascular endothelial functions using FMD and the factors that influence FMD.

## Methods

### Study design

This study was an open-label prospective single-arm trial. At baseline and 12 weeks following the initial treatment with trelagliptin (50–100 mg once per week), we evaluated the FMD, adiponectin level, asymmetric dimethylarginine (ADMA) level, mean carotid intima media thickness (CIMT), mean brachial-ankle pulse wave velocity (baPWV), fasting blood glucose level, hemoglobin A1c (HbA1c) level, serum lipid level, body weight, and body mass index (BMI).

### Subjects and setting

The subjects of this study were type 2 DM patients aged 20–74 years, who visited our outpatient department between July 2015 and June 2016. Eligibility criteria were patients with poor glycemic control (6.5% ≤ HbA1c < 10% or 110 mg/dL ≤ fasting blood glucose < 250 mg/dL) despite ≥8 weeks of diet and exercise therapies and patients with poor glycemic control despite treatment with oral antidiabetic drugs (excluding pioglitazone, which may have potent effects on vascular endothelial functions [7]). If patients used oral antidiabetic drugs before trelagliptin treatment, they were enrolled on the condition that treatment with such drugs had been initiated at least 8 weeks prior to the current treatment. Exclusion criteria were patients with a history of type 1 DM, insulin dependence, acute diabetic complications (e.g., diabetic ketoacidosis), secondary DM, pancreatitis, severe infections, alcoholism, severe mental disorders, and malignant tumors. Furthermore, patients with a fasting blood glucose level of ≥250 mg/dL, being treated with drugs that may affect the fasting blood glucose level (e.g., glucocorticoids), with hepatic dysfunction [with aspartate aminotransaminase (AST) and alanine aminotransferase (ALT) levels exceeding the upper limits of the institution’s baselines], with systolic blood pressure ≥160 mmHg, who had developed severe vascular diseases that required hospitalization within previous 6 months (e.g., stroke and myocardial infarction), who were or may be pregnant, with serum creatinine level of ≥1.5 mg/dL or creatinine clearance (Ccr) level of <30 mL/min, and who were deemed ineligible to participate by their physicians were excluded from the study [8–10].

### Treatment

Patients were treated with trelagliptin after a dose adjustment based on the renal function (i.e., when the serum creatinine level was ≥1.4 mg/dL in men and ≥1.2 mg/dL in women or Ccr level was <50 mL/min, the dose was reduced to 50 mg per week). When patients had no renal dysfunction, they were treated with a typical dose of 100 mg/week. It was decided to treat patients who discontinued the treatment during the study period (12 weeks) as dropouts. Even after the initiation of treatment with trelagliptin, the patients were encouraged to continue the diet and exercise therapies. In case patients concomitantly used oral antidiabetic drugs that were not approved by their physicians or reduced or increased the dose of or discontinued the study drug, they were treated as dropouts.

When the HbA1c level was ≥8% even at ≥8 weeks after the initiation of trelagliptin treatment, it was decided that the following measures would be taken at the physicians’ discretion: (1) in case of trelagliptin monotherapy, addition and gradual increase of another oral antidiabetic drug and (2) in case of concomitant treatment with trelagliptin and another oral diabetic drug, the latter would be gradually increased [11]. When two or more hypoglycemic events were observed after the initiation of trelagliptin treatment, it was decided to taper the dose of the antidiabetic drug other than trelagliptin (oral sulfonylurea drug, if any) at the physician’s discretion. In case patients developed hypoglycemia, it was decided to have them orally ingest glucose and check their blood glucose levels every 15 min until their hypoglycemic conditions disappeared. Hypoglycemia was defined as persisting hypoglycemic symptoms that improved by glucose uptake, glucagon treatment, or dietary intake and a glucose level of <50 mg/dL measured by self-monitoring the level or a blood test upon hospital arrival, regardless of the presence or absence of hypoglycemic symptoms [12].

### Outcomes

The primary outcome was change in FMD at baseline and 12 weeks after the initial trelagliptin treatment. The secondary outcome was the change between baseline and 12 weeks after trelagliptin treatment in the adiponectin level, ADMA level, mean CIMT, mean baPWV, fasting blood glucose level, HbA1c level, serum lipid level, and BMI.

### Flow-mediated dilatation

FMD was evaluated following the previously published study guidelines [13]. It was decided to ask the subjects to control their smoking, dietary intake, caffeine-containing fluid intake, and the intake of drugs with antioxidant action (e.g., vitamin C or vitamin E preparations) for 12 h before performing FMD. If the subjects were on regular oral medications, they would not take such medications on the day of FMD measurement that was performed from 9 to 11 am. The subjects would rest in a supine position for 15 min in a room maintained at 25 °C prior to the measurement. After inflating the blood pressure cuff wound around the right forearm to 50 mmHg above the systolic blood pressure for 5 min, the cuff was deflated to measure any changes in the right upper arm artery diameter. We measured the maximum diameter of the brachial artery, which dilated with increased blood flow within 2 min after the cuff release and expressed its ratio to the baseline artery diameter in percentage as FMD. The echocardiographic system and the probe used were the IE33 (Philips, Netherlands) and the L11-3 (Philips, Netherlands), respectively.

### Carotid intima media thickness and brachial-ankle pulse wave velocity

CIMT was measured with the patient in a supine position with the neck extended after 15 min of rest. The measurement was performed at sites 10 mm proximal to the arch of the common carotid artery using bilateral long-axis tomographic images. The mean CIMT was calculated as the mean value of the bilateral CIMT measurements. The value was expressed in millimeters [14]. The abovementioned echocardiographic system and probe were used for measurements. The measurement of baPWV was performed based on the PWV measured from volume pulse waves of the blood pressure cuffs wound around the upper arms and ankles using a blood pressure pulse wave measuring system (BP-203RPEIII, Omron Colin Co., Ltd., Japan). Measurements were performed after the patient rested in the supine position for 5 min. PWV is an index of pressure waves ejected from the heart to the aorta and recorded as pulse waves between two points of a vessel. This index is obtained by dividing the distance by the time difference between the two points and expressed as a unit of speed (cm/min). The mean baPWV was obtained as a mean value of the bilateral measurements [15].

### Measurement of other variables

Other variables that were recorded comprised age, sex, body weight, body mass index (BMI), smoking status, alcohol consumption, DM duration, fasting blood glucose level, serum C-peptide level, immunoreactive insulin (IRI) level, homeostasis model assessment (HOMA) index, β-cell function, HbA1c level, estimated glomerular filtration rate (eGFR), estimated daily urinary trace albumin amount, systolic blood pressure, diastolic blood pressure, low-density lipoprotein cholesterol (LDLC) level, high-density lipoprotein cholesterol (HDLC) level, triglyceride (TG) level, presence/absence of diabetic retinopathy, presence/absence of diabetic neuropathy, presence/absence of nephropathy, and presence/absence of cardiovascular diseases. The use of oral antidiabetic drugs before trelagliptin treatment was also recorded. BMI was calculated using the equation: body weight (kg)/height (m^2^). The question about smoking status was “Do you currently smoke habitually?” and options for its answer were Yes and No. The question regarding the alcohol consumption habit was “Do you regularly consume liquor (*sake*, beer, *shochu*, plum wine, etc.)?” and options for its answer were Yes and No. Blood sample data were prepared from collected venous blood. The blood IRI was measured using an immunoradiometric assay. In addition, the HOMA index was calculated using the following formula: fasting blood glucose [mg/dL] × fasting IRI [µU/mL]/405. The β-cell function was calculated using the following formula: 360 × fasting IRI (µU/mL)/fasting blood glucose (mg/dL) − 63 [16].

The serum lipids were determined using the standard enzymatic methods. We decided to measure and record the plasma glucose concentration using the glucose oxidase method, and the HbA1c levels using high-performance liquid chromatography based on the National Glycohemoglobin Standardization Program. The eGFR (mL/min/1.73 m^2^) was calculated using the following formula advocated by the Japanese Society of Nephrology [17]: eGFRcre = 194 × Cre^−1.094^ × age^−0.287^ (×0.739 for females). Traces of albumin in urine were measured using the turbidimetric method, and the estimated daily urinary albumin was measured by calculating the albumin–creatinine ratio (ACR) using the following formula: urinary trace albumin (mg/L)/urinary creatinine (mg/dL) × 100. The unit used was mg/gCr. Systolic and diastolic blood pressures were measured in a fasting state in the outpatient department and recorded as mmHg. The presence or absence of diabetic retinopathy was determined based on diagnoses by ophthalmologists. Moreover, the presence or absence of diabetic neuropathy was determined regardless of diagnosis of any of the following conditions: decreased Achilles tendon reflex, decreased vibratory sense in the lateral malleolus, and abnormal nerve conduction test results. The presence or absence of diabetic nephropathy was determined regardless of the diagnosis of nephropathy stage 2 or more advanced stages (ACR ≥ 30 mg/gCr). The presence or absence of cardiovascular disease was determined regardless of the patient currently suffering from or with a history of ischemic heart disease (e.g., angina pectoris, stroke, or arteriosclerosis obliterans). Adiponectin was measured using the latex agglutination method (LSI Medience Corporation, Japan) and ADMA using the high-performance liquid chromatography method (SRL, Inc., Japan).

### Safety

In this study, all potential adverse events were recorded as they occurred after the initiation of trelagliptin treatment. When such events were suspected to have causal relationships with trelagliptin treatment and the treatment was discontinued, the patients were considered to be dropouts.

### Statistical analysis

The population for analysis comprised subjects who could be followed up to 12 weeks after the initiation of treatment, excluding the dropouts. In addition to the patient backgrounds, the following endpoints were evaluated at baseline and 12 weeks after the trelagliptin treatment: FMD, adiponectin, ADMA, mean CIMT, mean baPWV, body weight, BMI, fasting blood glucose, HbA1c, eGFR, ACR, blood pressure, serum lipid, IRI, HOMA index, and β-cell function. For statistical analysis, Student’s *t* test or the Wilcoxon rank sum test was used. Furthermore, Pearson’s correlation analysis was used to examine the correlation between change in FMD and that in adiponectin level, ADMA level, HbA1c level, BMI, HOMA index, and β-cell function. The analysis was performed using Stata version 12.0 (StataCorp LP, College Station, TX, USA), with a significance level (two-sided) of P < 0.05. This study was conducted after the acquisition of written informed consent from the participating patients and upon the approval by the ethics committee of Ise Red Cross Hospital physicians. This study was registered with the UMIN Clinical Trials Registry System (Trial ID UMIN000018311).

## Results

A total of 30 patients who met the eligibility criteria were enrolled in this study. Of these, three patients were excluded as they discontinued oral drugs after the initiation of treatment. The remaining 27 patients were treated as the population for analysis in this study. Patient backgrounds at baseline are presented in Table 1. The mean age was 61.4 ± 10.8 years, and men represented 19 individuals of the population (70.3%). The duration of DM was 8.8 ± 7.6 years, BMI was 24.6 ± 3.2 kg/m^2^, and the HbA1c level was 7.4 ± 1.0%. The glomerular filtration rate was adequately maintained, with an eGFR of 82.7 ± 21.1 mL/min/1.73 m^2^. An investigation of the antidiabetic drugs used before trelagliptin treatment demonstrated that the biguanides were most commonly used (16 patients), followed by sulfonylureas (8 patients).

**Table 1:** Characteristics of patients included in the study

Age (years), mean (SD)	61.4 (10.8)
Male/Women (n)	19/8
BMI (kg/m²), mean (SD)	24.6 (3.2)
Body weight (kg), mean (SD)	64.9 (8.9)
HbA1c (%), mean (SD)	7.4 (1.0)
Fasting plasma glucose levels (mg/dL), mean (SD)	145.8 (35.9)
Duration of diabetes (years), mean (SD)	8.8 (7.6)
Alcohol consumption, %	36.0
Smoking, %	44.0
eGFR (mL/min/1.73 m^2^), mean (SD)	82.7 (21.1)
ACR (mg/gCr), mean (SD)	34.1 (54.6)
Systolic blood pressure (mmHg), mean (SD)	129.8 (8.0)
Diastolic blood pressure (mmHg), mean (SD)	79.7 (7.3)
LDLC(mg/dL), mean (SD)	120.5 (31.4)
HDLC (mg/dL) mean (SD)	53.2 (14.0)
TG (mg/dL), mean (SD)	134.3 (49.4)
Retinopathy, %	14.8
Neuropathy, %	31.8
Nephropathy, %	29.6
Cardiovascular diseases, %	3.7

Oral hypoglycemic agent	N (%)*
α-glucosidase inhibitors	0
Sulfonylureas	8 (29.6)
Biguanides	16 (59.2)
Glinide	0
SGLT2 inhibitors	0
Angiotensin-converting enzyme inhibitors/angiotensin II receptor blockers	9 (33.3)
Statin	10 (37.0)

*SD* standard deviation, *BMI* body mass index, *eGFR* estimated glomerular filtration rate, *ACR* albumin creatinine ratio, *HbA1c* hemoglobin A1c, *LDLC* low-density lipoprotein cholesterol, *HDLC* high-density lipoprotein cholesterol, *TG* triglyceride, cardiovascular diseases comprise angina pectoris, myocardial infarction, stroke, and arteriosclerosis obliterans; *SGLT2* sodium glucose cotransporter 2

* There is a case of duplicates

Table 2 shows changes in each parameter at baseline and 12 weeks after trelagliptin treatment. Trelagliptin treatment showed no significant changes in FMD (2.42 ± 2.7% at baseline vs. 2.66 ± 3.8% post-treatment, P = 0.785) and ADMA (0.41 ± 0.0 µg/mL at baseline vs. 0.40 ± 0.0 µg/mL post-treatment, P = 0.402). Trelagliptin treatment resulted in a significant increase of serum adiponectin level (7.72 ± 6.9 µg/mL at baseline vs. 8.82 ± 8.3 µg/mL at post-treatment, P = 0.002). Fasting plasma glucose levels were significantly (P = 0.033) decreased, whereas HbA1c, BMI, and blood pressure did not significantly change. LDLC was significantly (P = 0.001) decreased, whereas HDLC and TG did not significantly change. Mean ba-PWV was significantly (P = 0.045) decreased, whereas mean CIMT did not significantly change. IRI, HOMA index, and β-cell function did not significantly change.

**Table 2:** Changes in various parameters between baseline and 12 weeks after trelagliptin therapy

	Baseline	After 12 weeks	P value
FMD (%), mean (SD)	2.42 (2.7)	2.66 (3.8)	0.785
Adiponectin (μg/mL), mean (SD)	7.72 (6.9)	8.82 (8.3)	0.002
ADMA (μM/L), mean (SD)	0.41 (0.0)	0.40 (0.0)	0.402
BMI (kg/m^2^), mean (SD)	24.6 (3.2)	24.4 (3.0)	0.219
Body weight (kg), mean (SD)	64.9 (8.9)	64.5 (9.5)	0.266
HbA1c (%), mean (SD)	7.5 (1.0)	7.1 (0.9)	0.051
Fasting plasma glucose, mean (SD)	145.8 (35.9)	129.1 (36.4)	0.033
IRI (μU/mL), mean (SD)	14.3 (10.1)	14.5 (10.6)	0.913
HOMA index, mean (SD)	5.0 (4.0)	4.5 (4.2)	0.469
β-cell function, mean (SD)	69.0 (48.7)	88.3 (74.3)	0.193
eGFR (mL/min/1.73 m^2^), mean (SD)	82.7 (21.1)	79.8 (22.3)	0.062
ACR (mg/gCr), mean (SD)	39.7 (62.3)	34.7 (53.6)	0.244
Systolic blood pressure (mmHg), mean (SD)	129.8 (8.0)	129.9 (9.6)	0.945
Diastolic blood pressure (mmHg), mean (SD)	79.7 (7.3)	77.6 (8.8)	0.240
LDLC (mg/dL), mean (SD)	120.5 (31.4)	110.4 (28.7)	0.001
HDLC (mg/dL) mean (SD)	53.2 (14.0)	52.9 (12.9)	0.745
TG (mg/dL), mean (SD)	134.3 (49.4)	132.6 (54.5)	0.871
Mean CIMT (mm), mean (SD)	1.76 (1.3)	1.42 (0.5)	0.227
Mean ba-PWV (cm/s), mean (SD)	1633 (244.1)	1569 (217.1)	0.045

*SD* standard deviation, *FMD* flow-mediated dilatation, *ADMA* asymmetric dimethyl arginine, *BMI* body mass index, *eGFR* estimated glomerular filtration rate, *ACR* albumin creatinine ratio, *HbA1c* hemoglobin A1c, *LDLC* low-density lipoprotein cholesterol, *HDLC* high-density lipoprotein cholesterol, *TG* triglyceride, *IRI* immunoreactive insulin, *HOMA* homeostasis model assessment, *CIMT* carotid intima media thickness, *ba-PWV* brachial ankle pulse wave velocity

**Fig. 1:**
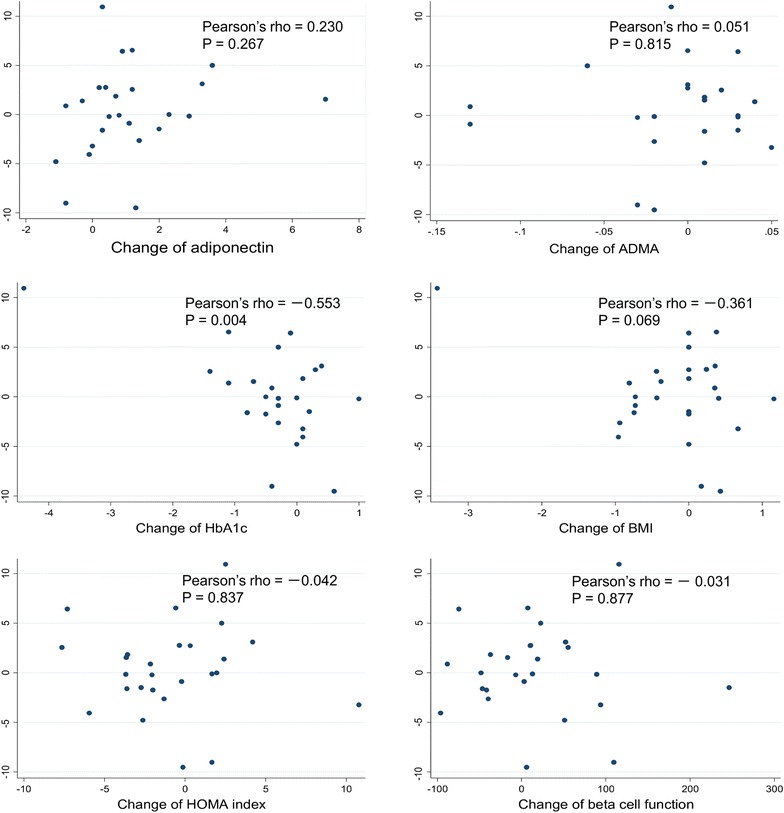
Univariate correlations between the changes of %FMD and those of adiponectin, ADMA, HbA1c, BMI, HOMA index, and β-cell function between baseline and after 12 weeks of trelagliptin therapy

Correlations between change in FMD and various parameters are presented in Fig. 1. Pearson’s correlation coefficient between change in FMD and that in the adiponectin level was 0.230 (P = 0.267) and in the ADMA level was 0.051 (P = 0.815) and that between change in FMD and that in HbA1c was −0.553 (P = 0.004). No significant correlation was observed between change in FMD and that in BMI, HOMA index, and β-cell function.

## Discussion

Trelagliptin treatment showed no significant changes in FMD and ADMA. On the other hand, trelagliptin treatment resulted in a significant increase of serum adiponectin level. Improvements in vascular endothelial function with DPP-4 inhibitor treatment have been reported in previous studies [8, 9]. Some researchers have reported that DPP-4 inhibitor treatment caused blood GLP-1 levels to increase, thereby improving postprandial vascular endothelium function [18, 19], and others have reported the likelihood of GLP-1 receptors existing in the vascular endothelium [20, 21]. Such reports have suggested the GLP-1 receptor-mediated direct improvement of vascular endothelial function. As we did not evaluate GLP-1 level and its influence on GLP-1 receptor in this study, we cannot mention these effects. On the other hand, although there was no significant decrease in HbA1c level after trelagliptin treatment in this study, a significant correlation between decrease in HbA1c level and increase in FMD. It was believed that vascular endothelial function improvement as a result of blood glucose level improvement may be possible. The fact that we did not observe any statistically significant differences in FMD reductions as a result of trelagliptin administration in this study may have been due to a lack of statistical power resulting from the small sample size. Further investigation with a larger sample size needs to be conducted in the future. Furthermore, in this study, the value of FMD was low. The subjects in this study had a long history of diabetes and the ratio of smoking was relatively high compared with that in the precedent study [8, 9]. We believe that a long history of diabetes and lack of instructions pertaining to smoking cessation resulted in a low FMD [22, 23].

Adiponectin, secreted primarily by adipose tissue, is known to have multifaceted effects, such as anti-inflammatory, antiarteriosclerotic, and antidiabetic activities [24, 25]. Adiponectin also increases the production of NO, a factor with vasodilating effects [26, 27]. Trelagliptin treatment in this study exhibited a significant increase in adiponectin, but a weak correlation between change in FMD and that in the adiponectin level. There have been reports on increased adiponectin levels after treatment with vildagliptin or sitagliptin or on the potential vascular endothelial function improvement mediated by increased adiponectin levels [8, 28, 29]. The present study was unable to clarify the mechanism of how trelagliptin treatment influences adiponectin levels. Other studies on the effects of vildagliptin or sitagliptin on adiponectin levels [11, 30] have reported that decrease in direct or indirect oxidative stress [31] or decrease in visceral fat may be involved in the mechanism of increase in adiponectin levels. As we did not evaluate oxidative stress or visceral fat in the present study, we cannot allude to this possibility via these mechanisms. On the other hand, one study reported that adiponectin levels did not significantly change after dipeptidyl peptidase-4 (DPP4) inhibitor treatment [32]. Further studies in the future are warranted to arrive at the conclusion of whether increase in adiponectin levels is a class or drug effect.

ADMA is a naturally occurring methylarginine derivative and an endogenous inhibitor of nitric oxide synthase, which plays the most important role in the control of the vascular endothelial functions [33]. Therefore, increased ADMA levels impair the NO production required for vasodilation, resulting in decreased vasodilation. Elevated ADMA levels are known as a predictive marker for the occurrence of cardiovascular diseases [34], and it is also known that DM patients have high ADMA levels [35]. In previous studies, researchers have reported that saxagliptin treatment increased NO levels in the vascular endothelium in animal experiments [36]. In addition, others have reported that vildagliptin treatment decreased ADMA levels [37]. Although the mechanistic details remain unknown, it is likely that DPP-4 inhibitor treatment improves the vascular endothelial functions by decreasing the ADMA levels, which in turn increases the NO levels. After trelagliptin treatment in this study, ADMA levels exhibited no significant changes, and a weak inverse correlation was observed between the change in FMD and that in the ADMA level. An inverse correlation between the ADMA level and FMD regarding vascular endothelial functions was reported in a previous study [38]. The lack of a statistically significant difference in ADMA decreases after trelagliptin administration may have been due to the sample size being too small. Therefore, this issue needs to be investigated again in the future with a larger sample size.

Although LDL levels significantly decreased after trelagliptin treatment in this study, HDL and TG levels and blood pressure did not considerably change. Previous studies have reported decreased LDL, increased HDLC, and decreased TG levels and improved blood pressure after alogliptin or sitagliptin treatment [39–41]. Reasons for some of the results obtained in this study being different from those of the previous studies include the enrollment of subjects for whom antihypertensive drugs or lipid-lowering drugs were administered and the likelihood of the power being too weak because of the small population of patients. In this study, body weight and BMI did not considerably change after trelagliptin treatment. Body weight reduction at 12 months after sitagliptin or vildagliptin treatment have been previously reported [30, 42]. Because the trial period was 3 months, we believe that in the future, it will be necessary to evaluate the long-term effects of this drug on body weight and BMI. Decrease tendency of HOMA index scores and increase tendency of β-cell function were also observed in this study. These results require careful interpretation because the fasting blood glucose level at baseline being ≥140 mg/dL meant that HOMA index and β-cell function results may not be completely reliable [16]. In this study, a statistically significant decrease was observed for mean baPWV after trelagliptin administration. To the best of our knowledge, there have been no previous reports showing a significant improvement of mean baPWV as a result of DPP-4 inhibitor administration. It has been reported that in addition to improved blood glucose levels, baPWV decreases might be affected by decreases in body weight and BMI or by increases in adiponectin [30, 43]. In the present study, decreasing tendencies, although not statistically significant, were noted in body weight and BMI following trelagliptin administration, and adiponectin increased significantly. These changes may have resulted in the statistically significant decrease observed for mean baPWV after trelagliptin administration.

It should be noted that this study has some limitations. The first potential issue is the patient characteristics of the subjects enrolled in this study. Compared with previous studies that examined the effects of DPP-4 inhibitors administered once per day on vascular endothelial functions, patients in this study had a longer duration of DM, and thus, potentially more advanced arteriosclerosis. It was likely that the effects of individual vascular endothelial function and improvement of vascular endothelial function with treatment intervention were relatively small in the patients comprised in this study [44, 45]. Therefore, we believe that further studies are necessary to examine patients with a shorter duration of DM. The second potential issue is factors potentially affecting vascular endothelial function, including antihypertensive drugs, lipid-lowering drugs, smoking, and caffeine intake. Although tests were performed without preparing these drugs and after providing thorough lifestyle-related advice, the effects of these factors may not have been eliminated. The third issue is the likelihood of the power being too weak because of the small population of patients. Further studies were considered necessary to examine a larger population of patients. The fourth issue is a problem of compliance. HbA1c did not change despite a significant reduction in fasting plasma glucose (FPG). We instructed subjects to take medicine regularly. However, the possibility of poor compliance was considered. The fifth issue involves the nonmeasurement of GLP-1 level and DPP-4 activity in this study. Therefore, we cannot determine whether trelagliptin has various effects based on the GLP-1 level and DPP-4 activity. Finally, the issue is that this study was intended to evaluate the vascular endothelial function at baseline and after trelagliptin treatment, with a study design that incorporated no control group. It will be necessary to conduct additional studies with a design that incorporates control groups to further verify the causal relationships.

## Conclusion

In this pilot study, trelagliptin treatment showed no significant changes in FMD. On the other hand, it was believed that trelagliptin treatment may increase serum adiponectin level. A limitation of this study is the small number of subjects examined and the absence of any control group. Therefore, we cannot apply our results to the general population of type 2 diabetes patients. Further studies, in particular two- or multi-arms trial, would be necessary to elucidate these points.

## Abbreviations

DM: diabetes mellitus
FMD: flow-mediated dilatation
ADMA: asymmetric dimethylarginine
NO: nitric oxide
DPP-4: dipeptidyl peptidase-4
GLP-1: glucagon-like peptide-1
CIMT: carotid intima media thickness
baPWV: brachial-ankle pulse wave velocity
HbA1c: hemoglobin A1c
BMI: body mass index
AST: aspartate aminotransaminase
ALT: alanine aminotransferase
Ccr: creatinine clearance
IRI: immunoreactive insulin
HOMA: homeostasis model assessment
eGFR: estimated glomerular filtration rate
LDLC: low-density lipoprotein cholesterol
HDLC: high-density lipoprotein cholesterol
TG: triglycerides
ACR: albumin–creatinine ratio

## References

[1] Fox CS, Golden SH, Anderson C, Bray GA, Burke LE, de Boer IH (2015). Update on prevention of cardiovascular disease in adults with type 2 diabetes mellitus in light of recent evidence: a scientific statement from the American Heart Association and the American Diabetes Association. Diabetes Care.

[2] Inaba Y, Chen JA, Bergmann SR (2010). Prediction of future cardiovascular outcomes by flow-mediated vasodilatation of brachial artery: a meta-analysis. Int J Cardiovasc Imaging.

[3] Rossi R, Nuzzo A, Origliani G, Modena MG (2008). Prognostic role of flow-me-diated dilation and cardiac risk factors in post-menopausal women. J Am Coll Cardiol.

[4] Pepine CJ (2009). The impact of nitric oxide in cardiovascular medicine: un-tapped potential utility. Am J Med.

[5] Forstermann U, Munzel T (2006). Endothelial nitric oxide synthase in vascular disease: from marvel to menace. Circulation.

[6] Nystrom T, Gutniak MK, Zhang Q, Zhang F, Holst JJ, Ahren B (2004). Effects of glucagon-like peptide-1 on endothelial function in type 2 diabetes patients with stable coronary artery disease. Am J Physiol Endocrinol Metab.

[7] Tsuchiya K, Akaza I, Yoshimoto T, Hirata Y (2009). Pioglitazone improves endothelial function with increased adiponectin and high-density lipoprotein cholesterol levels in type 2 diabetes. Endocr J.

[8] Kubota YK, Miyamoto M, Takagi G, Ikeda T, Kirinoki-Ichikawa S, Tanaka K (2012). The dipeptidyl peptidase-4 inhibitor sitagliptin improves vascular endothelial function in type 2 diabetes. J Korean Med Sci.

[9] Nakamura KN, Oe H, Kihara H, Shimada K, Fukuda S, Watanabe K (2014). DPP-4 inhibitor and alpha-glucosidase inhibitor equally improve endothelial function in patients with type 2 diabetes: EDGE study. Cardiovasc Diabetol.

[10] Mita TM, Katakami N, Shiraiwa T, Yoshii H, Onuma T, Kuribayashi N (2014). Rationale, design, and baseline characteristics of a clinical trial for prevention of atherosclerosis in patients with insulin-treated type 2 diabetes mellitus using DPP-4 inhibitor: the Sitagliptin Preventive study of Intima-media thickness Evaluation (SPIKE). Diabetol Metab Syndr.

[11] Hibuse T, Maeda N, Kishida K, Kimura T, Minami T, Takeshita E (2014). A pilot three-month sitagliptin treatment increases serum adiponectin level in Japanese patients with type 2 diabetes mellitus–a randomized controlled trial START-J study. Cardiovasc Diabetol.

[12] Kaku K, Watada H, Iwamoto Y, Utsunomiya K, Terauchi Y, Tobe K (2014). Efficacy and safety of monotherapy with the novel sodium/glucose cotransporter-2 inhibitor tofogliflozin in Japanese patients with type 2 diabetes mellitus: a combined Phase 2 and 3 randomized, placebo-controlled, double-blind, parallel-group comparative study. Cardiovasc Diabetol.

[13] Corretti MC, Anderson TJ, Benjamin EJ, Celermajer D, Charbonneau F, Creager MA (2002). Guidelines for the ultrasound assessment of endothelial-dependent flow-mediated vasodilation of the brachial artery: a report of the International Brachial Artery Reactivity Task Force. J Am Coll Cardiol.

[14] Lundby-Christensen L, Tarnow L, Boesgaard TW, Lund SS, Wiinberg N, Perrild H (2016). Metformin versus placebo in combination with insulin analogues in patients with type 2 diabetes mellitus-the randomised, blinded Copenhagen Insulin and Metformin Therapy (CIMT) trial. BMJ Open.

[15] Wu N, Cai X, Ye K, Li Y, He M, Zhao W (2014). Association between brachial-ankle pulse wave velocity and cardiac autonomic neuropathy in type 2 diabetes. Diabetol Metab Syndr.

[16] Matthews DR, Hosker JP, Rudenski AS, Naylor BA, Treacher DF, Turner RC (1985). Homeostasis model assessment: insulin resistance and beta-cell function from fasting plasma glucose and insulin concentrations in man. Diabetologia.

[17] The Japanese Society of Nephrology (2012). Clinical practice guidebook for diagnosis and treatment of chronic kidney disease 2012. Nihon Jinzo Gakkai Shi.

[18] Koska J, Schwartz EA, Mullin MP, Schwenke DC, Reaven PD (2010). Improvement of postprandial endothelial function after a single dose of exenatide in individuals with impaired glucose tolerance and recent-onset type 2 diabetes. Diabetes Care.

[19] Nikolaidis LA, Mankad S, Sokos GG, Miske G, Shah A, Elahi D (2004). Effects of glucagon-like peptide-1 in patients with acute myocardial infarction and left ventricular dysfunction after successful reperfusion. Circulation.

[20] Zhao T, Parikh P, Bhashyam S, Bolukoglu H, Poornima I, Shen YT (2006). Direct effects of glucagon-like peptide-1 on myocardial contractility and glucose uptake in normal and postischemic isolated rat hearts. J Pharmacol Exp Ther.

[21] Nystrom T, Gutniak MK, Zhang Q, Zhang F, Holst JJ, Ahren B (2004). Effects of glucagon-like peptide-1 on endothelial function in type 2 diabetes patients with stable coronary artery disease. Am J Physiol Endocri-nol Metab.

[22] Naka KK, Papathanassiou K, Bechlioulis A, Kazakos N, Pappas K, Tigas S (2012). Determinants of vascular function in patients with type 2 diabetes. Cardiovasc Diabetol.

[23] Kawano NN (2012). Association of endothelial and vascular smooth muscle dysfunction with cardiovascular risk factors, vascular complications, and subclinical carotid atherosclerosis in type 2 diabetic patients. J Atheroscler Thromb.

[24] Kishida K, Funahashi T, Shimomura I (2012). Molecular mechanisms of diabetes and atherosclerosis: role of adiponectin. Endocr Metab Immune Disord Drug Targets.

[25] Kishida K, Funahashi T, Shimomura I (2014). Adiponectin as a routine clinical biomarker. Best Pract Res Clin Endocrinol Metab.

[26] Margaritis M, Antonopoulos AS, Digby J, Lee R, Reilly S, Coutinho P (2013). Interactions between vascular wall and perivascular adipose tissue reveal novel roles for adiponectin in the regulation of endothelial nitric oxide synthase function in human vessels. Circulation.

[27] Lopez-Jaramillo P (2016). The role of adiponectin in cardiometabolic diseases: effects of nutritional interventions. J Nutr.

[28] Miyagawa K, Kondo T, Goto R, Matsuyama R, Ono K, Kitano S (2013). Effects of combination therapy with vildagliptin and valsartan in a mouse model of type 2 diabetes. Cardiovasc Diabetol.

[29] Sakr HF (2013). Effect of sitagliptin on the working memory and reference memory in type 2 diabetic Sprague-Dawley rats: possible role of adiponectin receptors 1. J Physiol Pharmacol.

[30] Shestakova MV, Suhareva OI, Chernova TO, Shmushkovich IA, Aleksandrov AA, Il’in AV (2013). A combination of dipeptidyl peptidase-4 inhibitor and metformin in the treatment of patients with type 2 diabetes mellitus: effective control of glycemia, weight, and quantitative body composition. Ter Arkh.

[31] Furukawa S, Fujita T, Shimabukuro M, Iwaki M, Yamada Y, Nakajima Y (2004). Increased oxidative stress in obesity and its impact on metabolic syndrome. J Clin Invest.

[32] Derosa G, Maffioli P, Salvadeo SA, Ferrari I, Ragonesi PD, Querci F (2010). Effects of sitagliptin or metformin added to pioglitazone monotherapy in poorly controlled type 2 diabetes mellitus patients. Metabolism.

[33] Cooke JP (2000). Does ADMA cause endothelial dysfunction?. Arterioscler Thromb Vasc Biol.

[34] Sibal L, Agarwal SC, Home PD, Boger RH (2010). The role of asymmetric dimethylarginine (ADMA) in endothelial dysfunction and cardiovascular disease. Curr Cardiol Rev..

[35] Cavusoglu E, Ruwende C, Chopra V, Poludasu S, Yanamadala S, Frishman WH (2010). Relation of baseline plasma ADMA levels to cardiovascular morbidity and mortality at two years in men with diabetes mellitus referred for coronary angiography. Atherosclerosis.

[36] Mason RP, Jacob RF, Kubant R, Walter MF, Bellamine A, Jacoby A (2011). Effect of enhanced glycemic control with saxagliptin on endothelial nitric oxide release and CD40 levels in obese rats. J Atheroscler Thromb.

[37] Cakirca M, Karatoprak C, Zorlu M, Kiskac M, Kanat M, Cikrikcioglu MA (2014). Effect of vildagliptin add-on treatment to metformin on plasma asymmetric dimethylarginine in type 2 diabetes mellitus patients. Drug Des Devel Ther.

[38] Boger RH, Bode-Boger SM, Szuba A, Tsao PS, Chan JR, Tangphao O (1998). Asymmetric dimethylarginine (ADMA): a novel risk factor for endothelial dysfunction: its role in hypercholesterolemia. Circulation.

[39] Ayaori M, Iwakami N, Uto-Kondo H, Sato H, Sasaki M, Komatsu T (2013). Dipeptidyl peptidase-4 inhibitors attenuate endothelial function as evaluated by flow-mediated vasodilatation in type 2 diabetic patients. J Am Heart Assoc.

[40] Seino Y, Fujita T, Hiroi S, Hirayama M, Kaku K (2011). Efficacy and safety of alogliptin in Japanese patients with type 2 diabetes mellitus: a randomized, double-blind, dose-ranging comparison with placebo, followed by a long-term extension study. Curr Med Res Opin.

[41] Mistry GC, Maes AL, Lasseter KC, Davies MJ, Gottesdiener KM, Wagner JA (2008). Effect of sitagliptin, a dipeptidyl peptidase-4 inhibitor, on blood pressure in nondiabetic patients with mild to moderate hypertension. J Clin Pharmacol.

[42] Derosa G, Carbone A, Franzetti I, Querci F, Fogari E, Bianchi L (2012). Effects of a combination of sitagliptin plus metformin vs metformin monotherapy on glycemic control, beta-cell function and insulin resistance in type 2 diabetic patients. Diabetes Res Clin Pract.

[43] Mahmud A, Feely J (2005). Adiponectin and arterial stiffness. Am J Hypertens.

[44] Tabatabaei-Malazy O, Fakhrzadeh H, Sharifi F, Mirarefin M, Arzaghi SM, Badamchizadeh Z (2015). Effect of metabolic control on oxidative stress, subclinical atherosclerosis and peripheral artery disease in diabetic patients. J Diabetes Metab Disord.

[45] Dalla Pozza R, Beyerlein A, Thilmany C, Weissenbacher C, Netz H, Schmidt H (2011). The effect of cardiovascular risk factors on the longitudinal evolution of the carotid intima medial thickness in children with type 1 diabetes mellitus. Cardiovasc Diabetol..

